# RBM20-Related Cardiomyopathy: Current Understanding and Future Options

**DOI:** 10.3390/jcm10184101

**Published:** 2021-09-11

**Authors:** Jan Koelemen, Michael Gotthardt, Lars M. Steinmetz, Benjamin Meder

**Affiliations:** 1 Department of Internal Medicine III, University of Heidelberg, 69120 Heidelberg, Germany; Jan.Koelemenoglu@med.uni-heidelberg.de; 2 DZHK (German Centre for Cardiovascular Research), Partner Site Heidelberg, 69120 Heidelberg, Germany; Lars.Steinmetz@stanford.edu; 3 DZHK (German Centre for Cardiovascular Research), Partner Site Berlin, 10117 Berlin, Germany; gotthardt@mdc-berlin.de; 4 Neuromuscular and Cardiovascular Cell Biology, Max Delbrück Center for Molecular Medicine in the Helmholtz Association, 13125 Berlin, Germany; 5 Charité-Universitätsmedizin Berlin, 13353 Berlin, Germany; 6 Department of Genetics and Stanford Genome Technology Center, Stanford University, Palo Alto, CA 94304, USA; 7 Genome Biology Unit, European Molecular Biology Laboratory (EMBL), 69117 Heidelberg, Germany

**Keywords:** dilated cardiomyopathy, RBM20, arrhythmia, heart failure, gene therapy, alternative splicing

## Abstract

Splice regulators play an essential role in the transcriptomic diversity of all eukaryotic cell types and organ systems. Recent evidence suggests a contribution of splice-regulatory networks in many diseases, such as cardiomyopathies. Adaptive splice regulators, such as RNA-binding motif protein 20 (RBM20) determine the physiological mRNA landscape formation, and rare variants in the RBM20 gene explain up to 6% of genetic dilated cardiomyopathy (DCM) cases. With ample knowledge from RBM20-deficient mice, rats, swine and induced pluripotent stem cells (iPSCs), the downstream targets and quantitative effects on splicing are now well-defined and the prerequisites for corrective therapeutic approaches are set. This review article highlights some of the recent advances in the field, ranging from aspects of granule formation to 3D genome architectures underlying RBM20-related cardiomyopathy. Promising therapeutic strategies are presented and put into context with the pathophysiological characteristics of RBM20-related diseases.

## 1. RBM20 Mutations Cause Highly Penetrant Cardiomyopathies

In 2009, the first case of RBM20-associated human cardiomyopathy was described. The report mentioned two large families with autosomal dominant dilated cardiomyopathy. Clinically, they became noticeable due to young age at diagnosis, heart failure and high mortality [1]. Since then, mutations in RBM20 were recognized as an important cause of cardiomyopathy and genotype-phenotype studies suggest many patients having a progressive and complicated clinical course [1,2,3,4,5,6].

**Figure 1:**
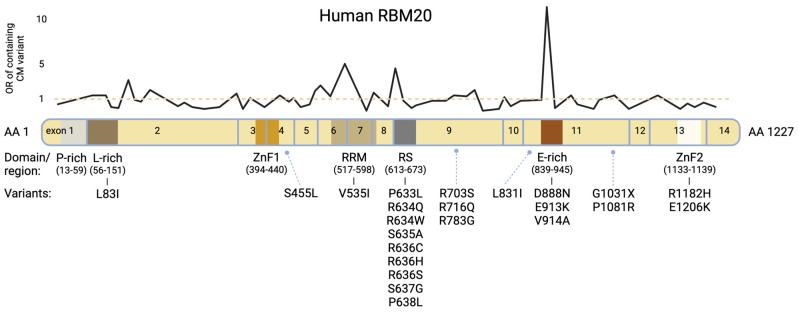
Modified from Parikh et al., 2019 [12]. Schematic protein structure of human RBM20 with corresponding exons. Variants shown in Table 1 are listed under the affiliated protein domains/regions. The black line chart displays the odds ratio (OR) for variant observation within the respective sections in a cardiomyopathy population vs. general population (Genome Aggregation Database [gnomAD]); underlying OR data derived from Parikh et al. [12]. Pathogenic sections predominantly concern the RS domain, the E-rich region and the RRM domain.

RBM20 regulates post-transcriptional splicing, particularly in sarcomeric, but also in other genes essential for myocardial homeostasis and calcium handling [1,6,7,8]. It is expressed in all striated muscles but highest in cardiac tissue [6]. The corresponding gene, RBM20, is located on the long arm of chromosome 10 and carries 14 exons. It encodes a 1227 amino acid protein containing two zinc finger domains, a glutamate-rich region, a leucine-rich region, an RNA-Recognition Motif (RRM)-type RNA binding domain and an arginine-/serine-rich region (RS-domain) (Figure 1) [9,10].

In patients with familial DCM, structured pedigree analysis is critical and genetic testing by sequencing of DNA commonly extracted from peripheral whole blood lymphocyte samples is recommended [11]. Pathogenic variants in RBM20 account for approximately 2–6% of the cases of familial DCM with noticeably early disease onset and clinically severe expression [2,12,13,14]. Figure 1 and Table 1 present reported variants with corresponding domains. Most patients carry heterozygous mutations, and the mode of inheritance is autosomal dominant [15]. Three protein regions were identified with high confidence for carrying pathogenic variants [12,15]. These are located at positions c.1601-1640 (exon 7, encoding the RRM-domain), c.1881-1920 (exon 9, encoding the highly conserved RS-domain) and c.2721-2760 (exon 11) [12,16,17]. In an international RBM20 patient registry, individuals with variants within these domains had a higher familial incidence of sudden cardiac death (SCD) and prevalence of personal history of arrhythmias than those with variants outside these hotspots or in genes such as Titin [12].

**Table 1:** RBM20 variants with corresponding exons and protein domains.

Domain	Mutation	Exon	Pathogenicity	Reference
Leu-rich-region	L83I	2	unknown	[18]
Other	S455L	4	unknown	[18]
RRM-domain	V535I	6	pathogenic	[6,16]
RS-domain	P633L	9	pathogenic	[19]
RS-domain	R634Q	9	pathogenic	[1,2,6,16]
RS-domain	R634W	9	pathogenic	[10,16]
RS-domain	S635A	9	pathogenic	[3,4,6,10]
RS-domain	R636C	9	pathogenic	[16]
RS-domain	R636H	9	pathogenic	[1,2,16,20,21]
RS-domain	R636S	9	pathogenic	[1,2,6,22]
RS-domain	S637G	9	pathogenic	[1,3,6,23]
RS-domain	P638L	9	pathogenic	[1,2,6,15,18]
Other	R703S	9	unknown	[18]
Other	R716Q	9	unknown	[6,16]
Other	R783G	9	pathogenic	[24]
Other	L831I	11	unknown	[18]
Glu-rich-region	D888N	11	unknown	[18]
Glu-rich-region	E913K	11	pathogenic	[2,25]
Glu-rich-region	V914A	11	pathogenic	[15]
Other	G1031X *	11	pathogenic	[10,18]
Other	P1081R	11	unknown	[18]
ZnF-2	R1182H	13	unknown	[13]
ZnF-2	E1206K	13	unknown	[18]

* non-sense mutation; all others are missense mutations.

Most of the disease-causing mutations have been identified within the RS domain [1,6,9,16,26]. The RS domain generally plays an important role in pre-mRNA splicing and regulating alternative splicing by modulating the binding and assembly of the spliceosome [27,28,29]. In the case of RBM20, it has been shown that Serines within the Arginine–Serine–Arginine–Serine–Proline (RSRSP) stretch of the RS-Domain are physiologically phosphorylated and serve as a critical part of the nuclear localization signal (NLS) [10]. Moreover, it is believed that mutations of any residues within the RSRSP stretch, possibly accompanied by aberrant phosphorylation, may cause RBM20 mislocalization, subsequently leading to altered nuclear splicing of the target pre-mRNAs [10]. Recently, a novel angle on molecular pathophysiology of RBM20-related cardiomyopathy was established, exemplified by findings from gene-edited RBM20-p.Arg636Ser pigs [22]. It was hypothesized that the disease, beyond missplicing due to loss-of-function mutations, could also be caused by gain-of-function mutations leading to dysregulated cytoplasmic RBM20 ribonucleoprotein (RNP) granule formation [22]. Subsequently, this granule formation might mediate myocardial insufficiency, which will be further discussed below [22].

Variants outside the RS-Domain commonly do not affect splicing activity in the same way as variants within the RS-Domain [10]. However, mutations within the conserved Glutamate-rich region have been associated with disturbed alternative splicing of Titin and DCM as well [2,10,18,25,26]. It seems that they affect protein stability and hence a partial loss-of-function can occur. An altered RBM20 function was also observed in the G1031X nonsense mutation, characterized by the loss of the second zinc-finger domain [10]. Interestingly, only a homozygous son of the characterized family showed symptoms while the heterozygous mother was asymptomatic [10]. Although variants affecting the RRM-domain have not been frequently identified as causes for human RBM20-related cardiomyopathy, it has been observed that the knockout of the RRM-domain by deletion of exons 6 and 7, both in homozygous and heterozygous KO mice, affected alternative splicing of RBM20′s target genes, such as *CAMK2D* and *LDB3* [30]. Additionally, a loss of the RRM-domain and RBM20′s C-terminus led to a significant reduction of RBM20′s splicing function also in human cell culture [31].

Accurate variant calling according to current ACMG criteria is a crucial yet challenging task in rare diseases. To assist in classifying novel RBM20 variants, the corresponding location within the protein presents a suitable indicator. In this context, Gaertner et al. showed that the ratio of ryanodine receptor 2 (*RYR2*)- and titin (*TTN*)-splice variation could be also used as a tool for the classification of uncharacterized RBM20 variants if heart tissue or iPSC are available [15]. The team used qRT-PCR to measure the expression of *RYR2*-splice variants containing an additional small exon, which was previously described [8]. This quantitative expression is then put into relation with the expression of the regular *RYR2*-splice form. They were able to show that this ratio is increased in the case of pathogenic RBM20-variants [15]. As controls, they used RYR2-isoform ratios from non-failing and DCM heart samples (both negative controls), along with positive controls derived from individuals carrying the proven pathogenic RBM20-p.Pro638Leu variant. Analogous to this, they investigated the ratio of TTN-N2B to total Titin as an additional classification tool and concluded that this ratio, on the contrary, is lowered in individuals carrying pathogenic RBM20-variants [15].

## 2. Model Systems to Dissect the Pathophysiology and Enable Therapeutic Studies

Currently, there are several in vitro and in vivo model systems available. As the first animal model served the rat, focusing mainly on RBM20-dependent splicing regulation of *TTN* [6,32]. Left ventricular dilation and electrical abnormalities were present in these RBM20-deficient rats, showing comparatively similar pathologies as RBM20-deficient humans [1,3,6,16].

RBM20 deficiencies are also studied in established mouse models. Mouse models show advantages due to their easy handling, however, LV dysfunction primarily occurs only after stress [30]. Gene editing by targeted disruption of specific exons or CRISPR/Cas9 system has been used to create RBM20 knockout models and RBM20-mutant knockin mice mimicking human-derived mutations [3,10,30,33,34]. In mice, mutations in RBM20 are associated with signs of arrhythmia, unlike knockout models, where the reduced or eliminated expression of RBM20 resulted in a less severe phenotype, exemplified by the comparison of homozygous RBM20-p.Ser637Ala mutants with the complete knockout of RBM20 [3]. Although, in both the mutant and knockout model, splicing activity of RBM20 targets was affected, there are several examples, where the mutant leads to an altered calcium handling and subsequently to arrhythmia, while homo- and heterozygous knockouts showed regular cardiac electrical activity [3,33]. This observation suggests that not only might changes in splicing activity be the driver of this disease, but also other underlying mechanisms that likely play important roles in the pathogenesis of RBM20-related cardiomyopathy exist [3,9,19,22,33].

In genome-edited pigs, hetero- or homozygous for RBM20 alleles encoding a pathogenic human-derived RBM20-variant, it was recently discovered that RBM20 RNP granules accumulated abnormally in the sarcoplasm of the myocytes [22]. As discussed above, these findings are of greater interest and were confirmed in myocardium and reprogrammed cardiomyocytes from DCM patients carrying the same pathogenic allele [22]. In vivo, the dysregulated RNP granule aggregation was more severe in the homozygous gene-edited pigs, which lead to myocardial insufficiency and fatal circulatory failure in many of these animals [22]. Consequently, the presence of these sarcoplasmic aggregations seems to play a relevant role in the pathophysiology of RBM20-related cardiomyopathy [3,15,22]. As such, stringent observation and examination of genome-edited pigs carrying pathogenic (RBM20) variants have the potential to provide critical insights into the pathomechanism and natural course of human DCM [22]. Additionally, the pig model can also serve as a valuable large animal model for succeeding therapy studies.

The investigation of splicing defects in human heart tissue and iPSCs provides a valuable toolkit for the translation of recent in vivo and -vitro findings into a human model system [4,8,15,19,22,35,36,37]. Patient-specific RBM20 mutant iPSCs and isogenic gene-corrected iPSCs have been established using CRISPR/Cas9, allowing the investigation of variant-specific RBM20-dependent pathomechanisms in a controlled setting [4,19,35,36,37]. These sorts of models also foster larger scale therapeutic drug screenings and provide a platform to study the advantages of novel technique applications, such as nanopore sequencing, in order to further investigate the genetic background of the disease [19,36,37].

## 3. Trafficking of RBM20 and Aggregation Formation

**Figure 2:**
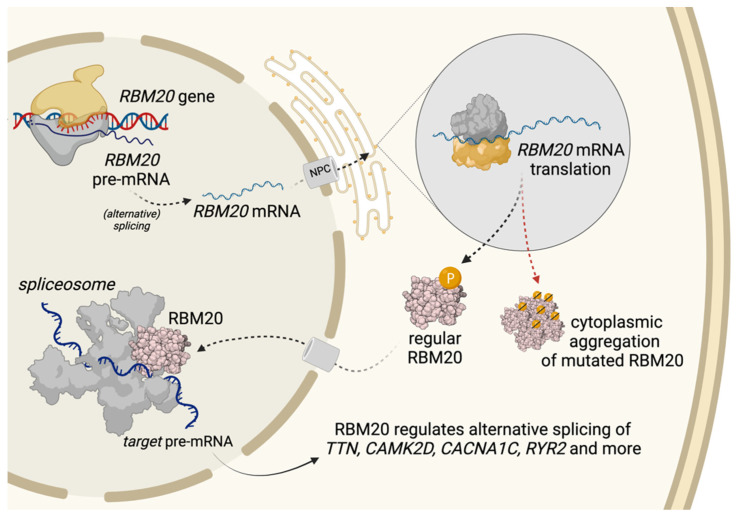
Modified from Schneider et al., 2019 [22]. RBM20 synthesis, trafficking and pathological cytoplasmic aggregation of RBM20-mutations. RBM20 is transcribed and its mRNA translocated through the nuclear pore complex (NPC) to the cytoplasm, where the RBM20 protein is translated. Regular RBM20 is transported back to the nucleus where it regulates alternative splicing as a part of the spliceosome. Disruption of nuclear transport processes in the presence of RBM20 mutations, however, may promote cytoplasmic protein aggregation, amongst other things by aberrant phosphorylation of the RS-Domain. This caused mislocalization may explain the altered splicing of target genes in the presence of RBM20 mutations.

Normal RBM20 synthesis and trafficking are outlined in Figure 2. Wild-type RBM20 is predominantly localized in the nucleus, being part of the spliceosome and functioning thus as a splicing cofactor. Analyses in C2C12 cells (mouse myoblast cell line) showed that RBM20 variants, such as RBM20-p.Pro638Leu or -p.Ser637Ala, lead to increased RBM20 aggregation within the cytoplasm [3,15]. Murayama et al. had shown similar results for murine p.Ser637Ala [10]. These findings from the murine models were confirmed not only in human explanted heart tissue but also in a recently published and already mentioned pig model [15,22]. Functionally, disruption of nuclear transport processes might lead to cytoplasmatic protein aggregation [38]. The mechanism responsible for this protein mislocalization is believed to be caused, for example, by aberrant phosphorylation of the RSRSP-stretch in the RS-domain [10,15]. Considering most patients are heterozygous carriers, nuclear concentrations of RBM20 remain lower, leading to functional deficiencies [15]. The described mislocalization also raises the idea that the dysregulated RNP granules could be interfering with cytoplasmic stress granules, possibly causing a detrimental cascade [3,22]. Upcoming results from currently ongoing investigations beyond nuclear splicing are eagerly awaited. The exploration and discovery of yet unidentified RBM20-partner proteins essential for its proper transportation to the nucleus and the interaction with the spliceosome and genome-foci represent additional directions of future research.

Not every RBM20-variant may lead to protein mislocalization [15]. The pathogenic RBM20-p.Val914Ala variant, for instance, showed no effect on RMB20′s nuclear localization, but on downstream splicing [15]. Rare variants leading to non-sense mediated RNA decay and haploinsufficiency may result in less severe cardiomyopathy, potentially due to missing substrate for aggregates [9,10,39]. It will be interesting to investigate the resulting molecular differences in pairwise comparisons.

## 4. Splicing Targets and Their Function

Until now, more than 30 validated splicing targets of RBM20 have been identified with high confidence [6,8,33]. Table 2 provides an overview of these target genes with the corresponding proteins and their functions. From a pathogenetic standpoint, some of the centrally involved targets include:

### 4.1. TTN

Titin is a giant protein embedded between the Z-disk and the M-band of the sarcomere [43]. It is crucial for myocardial contraction, elasticity and a key determinant for myocardial stiffness [43,44]. A loss of function in RBM20 causes DCM, often by missplicing of *TTN* and impaired Frank–Starling mechanism [6,8,25]. One of the primary underlying molecular pathomechanisms suspected of developing RBM20-associated DCM are the newly emerging giant titin isoforms [6,7,8]. Regularly, RBM20 mediates exon skipping of the Titin-PEVK region; when defective, exons remain and an embryonic, large Titin isoform is produced [6,7]. This titin isoform N2BA-G leads to reduced passive tension of the cardiac sarcomere and changed cardiac energetics, consequently being involved in the pathological heart chamber dilation [17,25,45]. To find out whether mutations in RBM20 are likely functional requires molecular diagnosis. As many patients would not want to have a biopsy taken for titin isoform expression analysis, this is currently done by using titin splice reporter assays [6,31].

### 4.2. RYR2

The regulation of muscle-specific splicing of *RYR2* (Ryanodine Receptor 2) by RBM20 is well established [6,7,8,15]. *RYR2* encodes a calcium ion channel mainly expressed in the heart, essential for calcium homeostasis and thus for proper myocardial contraction [46,47]. Cardiomyopathy-associated RBM20 variants cause overexpression of an *RYR2*-splice variant containing an additional small exon [8]. Clinically, arrhythmias are more prevalent in RBM-associated cardiomyopathy than in most other DCM forms [5,12]. It is current thinking that myocardial remodeling and fibrosis can trigger ventricular arrhythmias, but the modification of RYR2 may also propel arrhythmic instability and thus represents an appealing therapeutic target. Results from a study focusing on *RBM20* variants responsible for arrhythmogenic cardiomyopathy proposed that affected patients should be clinically viewed similar to other arrhythmogenic cardiomyopathy or catecholaminergic polymorphic ventricular tachycardia (CPVT), which is also caused by mutations in the *RYR2* gene [12].

### 4.3. CAMK2D

The serine/threonine-specific protein kinase CaMKII-δ (Ca^2+^/calmodulin-dependent protein kinase II) is involved in calcium homeostasis and reuptake in cardiomyocytes [48], which provides the link to RYR2 function. It was observed that mutations in RBM20 result in a CaMKII-δ isoform switch from the regular -δB and -δC isoforms to the -δA and -δ9 isoforms [33]. This alteration is responsible for an increased L-type Ca^2+^-current with intracellular Ca^2+^-overload and increases sarcoplasmic reticulum Ca^2+^ content in RBM20 KO cardiomyocytes, eventually promoting arrhythmogenesis [33]. It must be considered, however, that electrophysiological properties vary between rodents and humans, hence impeding the translation of findings in rodent to human-derived model systems [33]. Beyond altered Ca^2+^-handling, Zhang et al. proposed an alternative CaMKII-based pathomechanism. The team discovered that the CaMKII-δ9 isoform may mediate cardiomyopathy by causing cardiomyocyte DNA damage and cell death due to disrupted UBE2T (Ubiquitin-conjugating enzyme E2 T)-mediated DNA repair [49].

### 4.4. Identification of Novel Targets

Many of RBM20′s target genes listed in Table 1 were discovered by combining data from crosslink-immunoprecipitation (CLIP-seq) with transcriptome analysis of heart tissues from wild-type and RBM20-deficient rats as well as human heart failure patients [8]. Hence, this approach remains suitable for future identifications of other involved target genes.

Recently, transcriptome analysis using long-read sequencing in the presence of RBM20 mutations has discovered novel differentially expressed RBM20-dependent transcripts. This is exemplified by the discovery of two *IMMT* (inner membrane mitochondrial protein gene) isoforms that might play a role in RBM20 cardiomyopathy [37]. These results revealed that a more widespread adoption of long-read sequencing for transcriptome analysis could identify also further differentially expressed transcripts and provide context to previously identified alternatively spliced exons [37].

### 4.5. Interactions between RBM20s Targets

With the help of modern technologies such as Hi-C, RNA-seq and ATAC-seq researchers can investigate genome architecture in cardiomyocytes [50]. A network of gene loci from different RBM20 targets was identified, revealing an inter-chromosomal association and interaction between each target [50].

**Figure 3:**
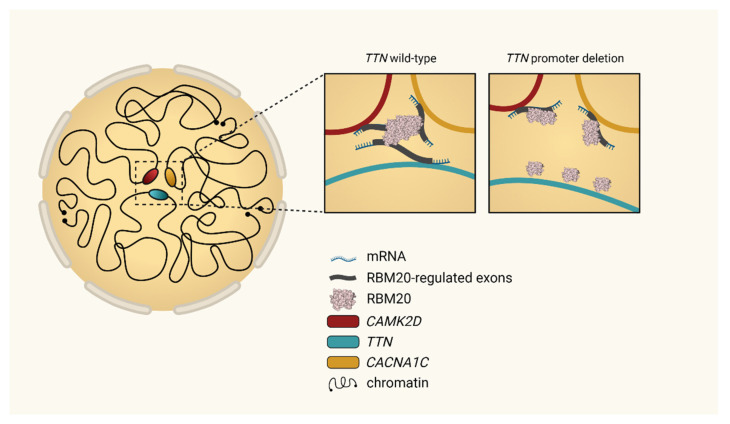
Modified from Bertero et al., 2019 [50]. Topological interchromosomal assembly of RBM20’s target genes. Model originally proposed by Bertero et al. to regulate local chromatin organization in human cardiomyocytes. During the differentiation of cardiomyocytes, heterochromatin becomes condensed, whereas major cardiac genes such as *TTN* move from the inactive (peripheral) to the active (central) compartment of the nucleus. Transcription of *TTN* forms foci of the splicing factor RBM20, resulting in a trans-interacting chromatin domain (TID) in which other RBM20 target genes are also involved. This mechanism drives alternative splicing of the emerging transcripts and can be interrupted by alteration of *TTN* transcription [50].

In human embryonic stem cells (hESC), the team discovered that *TTN* pre-mRNA, the best-studied RBM20-regulated transcript in the heart, binds and accumulates RBM20 foci near its genomic locus. This RBM20 accumulation mediates spatial proximity between the *TTN* locus and other inter-chromosomal RBM20 targets like *CAMK2D* or *CACNAC1C* (Figure 3). Changes in the topological assembly of the involved targets, for example, due to experimental deletion of the *TTN* promoter in hESC significantly reduced the spatial concentration of RBM20 genome-foci [50]. This is consecutively resulting in an altered RBM20-dependent alternative splicing activity of the other targets involved [50]. These novel perceptions indicate the existence of a cardiac-specific trans-interacting chromatin domain (TID) functioning in the sense of a splicing factory [50].

### 4.6. Interactions with Other Splice Regulators

Interactions of RBM20 with other splice regulators have been described in the literature. It was discovered recently that in titin splicing, PTB4 (polypyrimidine tract-binding protein isoform 4) counteracts the splice repressor activity of RBM20 [31]. PTB4 and RBM20 compete for the same motive on the 5′SS downstream of the alternative *TTN* exon, consequently regulating titin isoform expression [31]. These insights into the mechanistic interactions provide a basis for the future development of RBM20 modulators which adapt titin elasticity in cardiomyopathies [31].

Most recent studies suggest that RBM20 together with PTB4 also influences the splicing pattern of *FHOD3* (formin homology 2 domain containing 3) [51,52]. *FHOD3* was identified as an RBM20 target using RNA-seq, encodes a sarcomeric protein regulating actin dynamics in cardiac tissue and is associated with HCM and DCM [6,53,54,55,56]. The teams hypothesized that both splice regulators participate in the splice site recognition by competing with the snRNP (small nuclear ribonucleic particles) spliceosomal components, which determine the targets’ exon inclusion and exclusion outcome [51,52].

## 5. Clinical Presentation and Risk Management

Pathogenic RBM20-variants are associated with a clinically aggressive form of DCM or left-ventricular non-compaction (LVNC; RBM20 variants are detected in ~1% of LVNC cases) [17,57,58]. In our Cardiomyopathy Center, we also observed different families with Hypertrophic Cardiomyopathy (HCM) carrying pathogenic RBM20 variants. The onset of the disease is around the mid-fourth to fifth life decade and might become apparent due to heart failure or arrhythmia [5]. Male patients generally show a more severe clinical course including significantly earlier disease onset and higher adverse event rates (heart transplantation (HTx), sudden cardiac arrest (SCA), ventricular tachycardia) [2]. In progressed stages, heart transplantation and assist devices (LVAD) are often required [1,2,5,12,13,16,18,33].

The likelihood of developing life-threatening arrhythmias is relatively high, resulting in a poor prognosis if an implanted defibrillator does not protect the patient. Of the RBM20 mutation carriers, 30% developed conduction system disorders associated with a high risk of malignant arrhythmias [5]. Data from the international RBM20 patient registry provide a valuable overview of the relative risk for RBM20 mutation carriers compared to DCM of other etiology (Table 3). Odds ratios are comparable to arrhythmogenic *LMNA*-mutation-induced DCM and significantly higher than in idiopathic or *TTNtv*-mediated DCM [12]. In the study cohort, ICD discharge and sudden cardiac arrest were observed in several mutation carriers even before the onset of left ventricular dysfunction [12]. This requires careful risk stratification in RBM20-associated cardiomyopathy and discussion of primary preventive ICD implantation.

Of patients with RBM20 cardiomyopathy, 12% receive heart transplantation due to end-stage heart failure at a remarkably young mean age of 28 years, occurring significantly earlier than in other DCM genotypes [5]. A study in a Danish population observed transplantation rates of overall 21% (34% in males) [2]. Similar results were demonstrated by Gaertner et al. in 2020, where family members carrying the pathogenic RBM20-p.Pro638Leu variant had an average event-free (HTx, LVAD or death) survival time of 28 years [15].

## 6. Current Treatment Concepts in RBM20 Patients

### 6.1. Treatment of Heart Failure

Patients with RBM20-mediated DCM often present with systolic heart failure. Depending on the symptomatic extent of heart failure and systolic dysfunction, pharmacological treatment is carried out according to current therapeutic guidelines for acute and chronic heart failure [11,59,60]. ACE inhibitors, beta-blockers and mineralocorticoid antagonists work well in RBM20-related heart failure. The introduction of ARNI (sacubitril/valsartan) and SGLT2-inhibitors are other promising treatment options. RBM20-specific pharmacological treatments are not established.

In specialized centers worldwide, treatment with heart failure medications is advised already in the early stages of systolic dysfunction if the pathogenic mutation is known in the patient and deterioration is foreseen. These expert decisions are based on individual basis and experience and cannot be generalized at the moment.

### 6.2. ICD-Therapy

Current guidelines do not treat RBM20 cardiomyopathy separately from other causes of DCM. Considering available data on the risk of suffering from severe arrhythmias (Table 3), implantation of a primary prophylactic cardiac defibrillator should be discussed for individuals carrying proven pathogenic RBM20-variants [2,12]. In order to provide the same evidence that currently exists for the highly arrhythmogenic *LMNA/C* mutations [13,61,62,63], further studies are needed for generally recommending a primary prophylactic ICD implantation in consideration of the RBM20-mutation status.

### 6.3. Heart Transplantation and Assist Devices

(Temporary) support in severe heart failure can be reached by implanting a left-ventricular assist device. Heart transplantation is considered a last resort, yet indispensable due to the potentially severe clinical course of RBM20-related cardiomyopathy. As mentioned above, in a recent meta-analysis, the mean age of HTx in patients with RBM20-induced DCM was only 28 years [5]. Unlike many other genetic myopathies affecting sarcomere function, RBM20 defects mainly affect the myocardium and do not lead to skeletal myopathy [6]. This is an important aspect of considering patients for heart transplantation.

## 7. Future Therapeutic Options

Established generic therapies for heart failure and SCD-prevention are powerful tools to improve the prognosis of patients. However, experimental compounds or repurposing of existing drugs are attractive strategies for identifying tailored and specific RBM20-therapies. Therefore, suitable model systems are needed and stringent, and data-guided translation into the clinics is required to prevent harm by self-treatment of affected patients with experimental compounds.

### 7.1. RBM20 Upregulation

In 2020, Briganti et al. created an RBM20-deficient DCM model that recapitulates mRNA splicing and contractile defects of the disease using CRISPR/Cas9 in iPSC [19]. By bioinformatics screens, interesting compounds associated with RBM20 expression were identified and investigated functionally. The teams showed that the application of retinoic acid (ATRA), an active metabolite of vitamin A, upregulates RBM20 expression in murine and human-derived iPSC, partially reverting the splicing and contractile defects caused by pathogenic RBM20 variants [19]. This effect could be primarily shown in heterozygous but also in homozygous mutants, while not in the complete knockouts suggesting that the beneficial effect of ATRA remains dependent on residual RBM20 protein activity [19]. The transfer to the clinics requires functional proof in animal models and a careful clinical trial design since ATRA also has potential toxic side effects in high doses. Additionally, the RBM20-RNP-granules could be, as already mentioned, aggravated through upregulation of the RBM20-locus, potentially leading to (long-term) negative consequences.

### 7.2. RBM20 Downregulation

Besides the harm caused by RBM20 mutations, beneficial effects of RBM20 loss-of-function have been described as well. In heterozygous RBM20 KO-mice with an in-frame RRM deletion, a more compliant, large titin was identified, possibly leading to improved diastolic filling [30]. However, it must be considered that the impaired Frank–Starling mechanism in this scenario might outweigh the potentially beneficial effects [30].

With the development of an in vitro splice reporter assay in HEK293 cells, it was for the first time possible to screen > 34,000 small molecules for the potential treatment of diastolic dysfunction [64]. The study identified cardenolides as inhibitors of RBM20 dependent splicing, leading in the case of titin to the exclusion of PEVK exons. This again affected titin isoform expression, subsequently improving diastolic filling. These findings show that RBM20 downregulation might be helpful for future treatments of diastolic dysfunction and should be further investigated.

With the introduction of these sorts of splice reporter assays, it also remains exciting to see whether other small compounds will be identified in the future, providing novel therapeutic strategies through the regulation of RBM20 expression.

### 7.3. Ca^2+^-Modulation

RBM20-dependent Ca^2+^-homeostasis was first studied in knockdown mouse embryoid bodies [65]. Experimental data show that RBM20 loss-of-function in RBM20 KO-mice disturbed the Ca^2+^-handling resulting in arrhythmogenic Ca^2+^-releases/spikes from the sarcoplasmic reticulum [33]. This alteration was not only observed in homozygous but also in heterozygous mice [33]. They also discovered that the L-Type calcium channel (LTCC) activity was increased in RBM20 KO-mice, possibly due to an altered splicing of *CAMK2D* and *CACNAC1C*, likewise causing a more arrhythmogenic phenotype [33]. Wyles et al. investigated the Ca^2+^-homeostasis in an RBM20 patient-derived iPSC model and observed improved Ca^2+^-handling during a ß-adrenergic stress test by pretreatment with either the beta-blocker carvedilol or the L-Type-calcium-antagonist verapamil [36]. Based on the RBM20 KO-mice model, considering verapamil as a specific treatment option of arrhythmogenic dilated RBM20-cardiomyopathy was the subject of another recent study [33]. However, human data are limited and potential benefits from beta-blocker therapy would be withdrawn in this case. At the same time, calcium-antagonists have proven to be unsuccessful in the treatment of “common” heart failure.

Further evidence from current studies in iPSC and prospectively in a controlled clinical trial, is curiously awaited.

### 7.4. Gene Editing

As for many other genetic disorders, CRISPR/Cas9-based gene editing provides a powerful tool to investigate and possibly repair genetic abnormalities seen in RBM20-related cardiomyopathy. With the help of this technique, knockout and knockin models are already established in mouse, pig, hESC and iPSC models, and provide the basis for the identification and development of novel genetic treatment strategies [3,4,19,22,35,50]. Until now, CRISPR/Cas9-based repair approaches of pathogenic RBM20-variants are still in very early stages but represent an exciting and innovative field of research for upcoming investigations.

## 8. Conclusions

RBM20-related cardiomyopathy is an excellent example of how structured, collaborative clinical and experimental research can further our knowledge to a degree where clinical decision-making is considerably improved, and molecularly guided therapeutic options are emerging. With an armamentarium of potential options, the chances are high that breakthrough developments will change the course of this disease group in the near future.

## Figures and Tables

**Table 2:** Modified from Lennermann et al., 2020 [40]. List of RBM20 target genes with corresponding protein and function.

Gene	Encoded Protein	Function	References
*APTX*	Aprataxin	DNA repair	[6]
*CACNA1C*	Calcium channel, voltage-dependent, L-type, alpha 1C sub-unit	sub-unit of the L-type calcium channel	[6]
*CAMK2D*	Calcium/calmodulin-dependent protein kinase II delta	Serine/threonine kinase; regulates many cardiac proteins through phosphorylation	[6,33]
*CAMK2G*	Calcium/calmodulin-dependent protein kinase II Gamma	Serine/threonine kinase; regulates many cardiac proteins through phosphorylation	[6]
*DAB1*	Disabled-1	Neuronal development	[6]
*DNM3*	Dynamin-3	Actin-membrane budding	[6]
*DST*	Dystonin	Adhesion junction plaque protein	[8]
*DTNA*	Dystrobrevin alpha	Part of the dystrophin-associated complex linking ECM and cytoskeleton	[6]
*ENAH*	Protein-enabled homolog	Actin-associated	[8]
*FHOD3*	Formin homology 2 domain-containing 3	Sarcomeric assembly	[6]
*FNBP1*	Formin-binding protein 1	Actin cytoskeleton regulation	[6]
*GIT2*	G protein-coupled receptor kinase interactor 2	Cytoskeletal dynamics	[6]
*IMMT*	Inner membrane mitochondrial protein	Part of the mitochondrial inner membrane complex	[8,37]
*KALRN*	Kalirin	Serine/threonine protein kinase	[6]
*KCNIP2*	KV channel-interacting protein 2	Sub-unit of voltage-gated potassium channel complex	[6]
*LDB3*	LIM domain binding 3	Sarcomeric stabilization	[6,8]
*LMO7*	LIM domain only protein 7	-	[8]
*LRRFIP1*	Leucine-rich repeat flightless-interacting protein 1	Transcriptional repressor	[8]
*MECP2*	Methyl CpG–binding protein 2	Transcriptional regulator; highly expressed in neuronal cells	[6]
*MLIP*	Muscular-enriched A-type laminin-interacting protein	Interacts with lamin A/C; potentially involved in cardiac homeostasis	[8]
*MTMR1*	Myotubularin-related protein 1	-	[6]
*MYH7*	Myosin heavy chain 7	Cardiac slow twitch myosin heavy chain beta isoform; muscle contraction	[8]
*MYOM1*	Myomesin-1	Sarcomeric; links titin and thick filament	[8]
*NEXN*	Nexilin	Actin-associated; DCM-associated	[8]
*NFIA*	Nuclear factor I A	Transcription factor	[6]
*NPRL3*	Nitrogen permease regulator-like 3	Inhibits mTORC1; necessary for cardiovascular development	[6,41]
*NTRK3*	Tropomyosin receptor kinase C	Neutrophin-3-receptor	[6]
*OBSCN*	Obscurin	Sarcomeric signaling	[8]
*PDLIM3*	PDZ and LIM domain protein 3	Binds alpha actinin-2; relevant for right ventricular function	[8]
*PDLIM5*	PDZ and LIM domain protein 5	LIM domain protein; protein-protein interaction	[6,42]
*PLEKHA5*	Pleckstrin homology domain-containing family A member 5	-	[6]
*RALGPS1*	Ral GEF with PH domain- and SH3-binding motif 1	-	[6]
*RTN4*	Reticulon 4	Neurite outgrowth inhibitor in the central nervous system	[8]
*RYR2*	Ryanodine receptor 2	Calcium receptor in the SR; allows release of Ca^2+^ into the cytosol	[8]
*SEMA6D*	Semaphorin 6D	Neuronal regulation	[6]
*SH3KBP1*	SH3 domain-containing kinase-binding protein 1	-	[6]
*SLC38A10*	Putative sodium-coupled neutral amino acid transporter 10	Sodium-dependent amino acid/proton antiporter	[6]
*SORBS1*	Sorbin and SH3 domain-containing 1	Cytoskeletal formation	[6]
*SPEN*	Msx2-interacting protein	Hormone inducible transcriptional repressor	[6]
*TNNT2*	Cardiac troponin T	Part of the cardiac troponin complex regulating muscle contraction dependent on calcium	[8]
*TPM1*	Tropomyosin alpha-1 chain	Cytoskeletal; contraction	[6]
*TRDN*	Triadin	Forms a complex with RyR and CASQ2; calcium release from the SR	[6]
*TTN*	Titin	Sarcomeric spring; compliance of the heart	[6,8]
*UBE2F*	Ubiquitin-conjugating enzyme E2 F (putative)	-	[6]
*ZNF451*	E3 SUMO-protein ligase ZNF451	Protein sumoylation	[6]

**Table 3:** Modified from Parikh et al., 2019 [12]. *RBM20*-related cardiomyopathy has a highly arrhythmogenic phenotype.

	RBM20-CM vs. DCM Odds Ratio (CI; *p*-Value)	RBM20-CM vs. *TTNtv*-CM Odds Ratio (CI; *p*-Value)	RBM20-CM vs. *LMNA*-CM Odds Ratio (CI; *p*-Value)
Evidence of sustained VA *	14.7 (6.0–36.0; *p* < 0.001)	27.3 (3.4–223.0; *p* < 0.001)	1.2 (0.6–2.4; *p* = 0.65)
Family history of SCA **	5.9 (3.1–11.2; *p* < 0.001)	6.2 (2.6–14.5; *p* < 0.001)	1.4 (0.6–2.8; *p* = 0.46)

Odds ratios for sustained ventricular arrhythmias (VA) and family history of sudden cardiac arrest (SCA) for RBM20-CM cohorts compared to DCM, titin truncating variants (*TTNtv*)-CM and Lamin A/C (*LMNA*)-CM cohorts. * Sustained VA is defined as sustained ventricular tachycardia or ventricular fibrillation on monitoring for DCM, *TTNtv* and *LMNA* and as SCA or ICD discharge for RBM20. ** SCA in RBM20 index cases only. CI = confidence interval.
